# Circular-polarized-light-induced spin polarization characterized for the Dirac-cone surface state at W(110) with C_2*v*_ symmetry

**DOI:** 10.1038/s41598-018-28693-3

**Published:** 2018-07-11

**Authors:** K. Miyamoto, H. Wortelen, T. Okuda, J. Henk, M. Donath

**Affiliations:** 1 Hiroshima Synchrotron Radiation Center, Hiroshima University, 2-313 Kagamiyama, Higashi-Hiroshima, 739-0046 Japan; 2 Westfälische Wilhelms-Universität Münster, Physikalisches Institut, Wilhelm-Klemm-Straße 10, 48149 Münster, Germany; 3 Martin-Luther-Universität Halle-Wittenberg, Institut für Physik, Von-Seckendorff-Platz 1, 06120 Halle, Germany

## Abstract

The C_2*v*_ surface symmetry of W(110) strongly influences a spin-orbit-induced Dirac-cone-like surface state and its characterization by spin- and angle-resolved photoelectron spectroscopy. In particular, using circular polarized light, a distinctive **k**-dependent spin texture is observed along the $\overline{{\boldsymbol{\Gamma }}{\boldsymbol{H}}}$ direction of the surface Brillouin zone. For all spin components P_x_, P_y_, and P_z_, non-zero values are detected, while the initial-state spin polarization has only a P_y_ component due to mirror symmetry. The observed complex spin texture of the surface state is controlled by transition matrix element effects, which include orbital symmetries of the involved electron states as well as the geometry of the experimental set-up.

## Introduction

Topological insulators (TI) and Rashba systems attract great attention with regard to manipulation and generation of spin currents without magnetic field^1–3^. The lack of space-inversion symmetry at the surface of these materials leads to spin-polarized surface states driven by the spin-orbit interaction. The electron spin is locked to its crystal momentum, forming a unique helical spin texture. As a consequence of spin-orbit interaction, the spin-polarized surface state, by hybridization, consists of both spin-up and spin-down components with different weight of their partial wave functions^4–7^, except for the special case of wavevectors with C_3_ symmetry^8–11^.

The spin mixture causes spin entanglement of photoelectrons in topological materials as well as Rashba systems. Consequently, when spin-up and spin-down components in such initial bands are simultaneously excited by photons with a coherent mixture of *s*- and *p*-polarized light or by circular polarized light, a spin component other than that in the initial state is caused by the superposition of complex transition matrix elements.

Recently, by utilizing the above-mentioned effect, a fascinating idea was proposed: the manipulation and control of spin polarization of the photoelectron signal by a proper selection of the light polarization, experimental geometry and photon energy^5,6,12–19^. These phenomena are promising in view of the material-light-spin relationship for potential applications in optospintronics devices with multiple functionalities. So far, most studies of the photoelectron spin are restricted to surfaces with *C*_3*v*_ symmetry or to cases with normal electron emission^20–24^.

A surface state on W(110) within a spin-orbit-induced symmetry gap^25–29^ shows Dirac-cone-like dispersion with a spin texture reminiscent of a topological surface state (TSS)^30^. The surface state is strongly influenced by the twofold symmetry (C2*v*) of the crystal surface: it shows a flattened dispersion behavior along the $\overline{{\rm{\Gamma }}N}$ line of the surface Brillouin zone and a linear dispersion along $\overline{{\rm{\Gamma }}H}$ and $\overline{{\rm{\Gamma }}S}$^31–33^. Only $\overline{{\rm{\Gamma }}N}$ and $\overline{{\rm{\Gamma }}H}$ possess mirror planes. Based on several theoretical and experimental studies, along $\overline{{\rm{\Gamma }}H}$, the surface state exhibits predominant Σ_1_ (${d}_{{z}^{2}}$) and Σ_3_ (*d*_*zx*_) symmetry (single-group representation) with minor Σ_2_ (*d*_*xy*_) and Σ_4_ (*d*_*yz*_) contributions. We have demonstrated by angle-resolved photoelectron spectroscopy (ARPES) using linear *p*- and *s*-polarized light^4^ that the spin polarization depends on the orbital symmetry in agreement with theoretical calculations^7^. Moreover, recent theoretical research has shown that this surface state is topologically protected by mirror symmetry along $\overline{{\rm{\Gamma }}H}$. Therefore, W(110) is called a topological crystalline transition metal^34^.

In this work, we use spin-resolved ARPES with left and right circular polarized light to measure the three spin-polarization components of photoelectrons emitted from the Dirac-cone like surface state along $\overline{{\rm{\Gamma }}H}$ on W(110) with C2*v* symmetry. We show that the observed complex spin-polarization texture as a function of momentum can be explained by multiple contributions of the dipole-transition matrix element for the given highly symmetric experimental geometry. The observed spin polarization of the photo-emitted electrons is not only determined by the *intrinsic* spin polarization, i.e. the spin polarization of the initial state under investigation, but also by *extrinsic* spin polarization, which is induced and controlled by experimental parameters. In detail, the latter is given by phase differences between complex partial transition matrix elements, which contain the orbital components of initial and final states as well as experimental parameters such as the crystal symmetry, light polarization and its angle of incidence. In short, using the example of the Dirac-like surface state on W(110), we show how the spin polarization of photo-emitted electrons can be controlled experimentally.

## Results and Discussion

**Figure 1:**
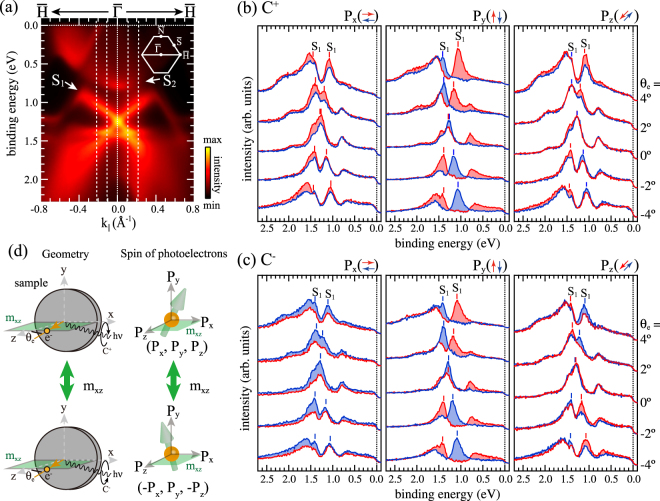
(**a**) Spin-integrated ARPES data taken with right circular polarized light of *hν* = 43 eV. (**b**) Spin-resolved energy distribution curves (EDCs) for all three spin-polarization components presented for selected emission angles *θ*_*e*_ along the $\overline{{\rm{\Gamma }}H}$ line, obtained for right circular polarized light (C^+^) of *hν* = 43 eV. The spin-dependent intensities are presented as red and blue lines. (**c**) Same as (**b**) but for left circular polarized light (C^−^). The dashed lines in (**a**) correspond to the EDCs given in (**b**) and (**c**). (**d**) Experimental geometry including the detection plane, which coincides with the mirror plane *m*_*xz*_ of the crystal.

Figure 1(a) shows spin-integrated ARPES data of W(110) along $\overline{{\rm{\Gamma }}H}$, obtained with right circular polarized light (C^+^) of photon energy *hν* = 43 eV. In agreement with established results for linear polarized light^4,31^, there are two characteristic surface states (*S*_1_ and *S*_2_) and bulk continuum states at binding energies *E*_*B*_ higher than 1.45 eV. In this paper, we only discuss the surface state *S*_1_ with clear Dirac-cone-like dispersion and a crossing point at *E*_*B*_ = 1.25 eV at $\overline{{\rm{\Gamma }}}$.

Figure 1(b,c) show spin-ARPES data as energy distribution curves (EDC’s) for selected emission angles *θ*_e_ along $\overline{{\rm{\Gamma }}H}$, excited by right (C^+^) and left (C^−^) circular polarized light. Left, middle and right columns present spin-ARPES results for P_x_, P_y_ and P_z_, respectively. Spin-up and spin-down intensities are plotted as red and blue solid lines, respectively.

At first, we will discuss spin-EDC’s for P_y_ in the middle panel of Fig. 1(b). A sharp peak *S*_1_ in the spin-up (spin-down) channel is located at *E*_*B*_ = 1.42 eV (1.05 eV) for *θ*_e_ = −4° and moves to lower (higher) *E*_*B*_ with increasing *θ*_e_. At *θ*_e_ = 0°, the bands cross, yet with spin-dependent intensities. This behavior is reminiscent of our previous results obtained for *p*-polarized light^4^. Moreover, according to the middle panel of Fig. 1(c), the observed spin features remain unchanged upon switching the circular polarization.

For P_z_ in Fig. 1(b), the spin texture of *S*_1_ is similar to P_y_ but with reduced spin difference. For P_x_, the spin-up intensities always exceed the spin-down intensities for both the upper and lower part of the Dirac cone. Note that, in contrast to P_y_, P_x_ and P_z_ switch sign upon reversing the circular polarization of the light (see Fig. 1(c)).

**Figure 2:**
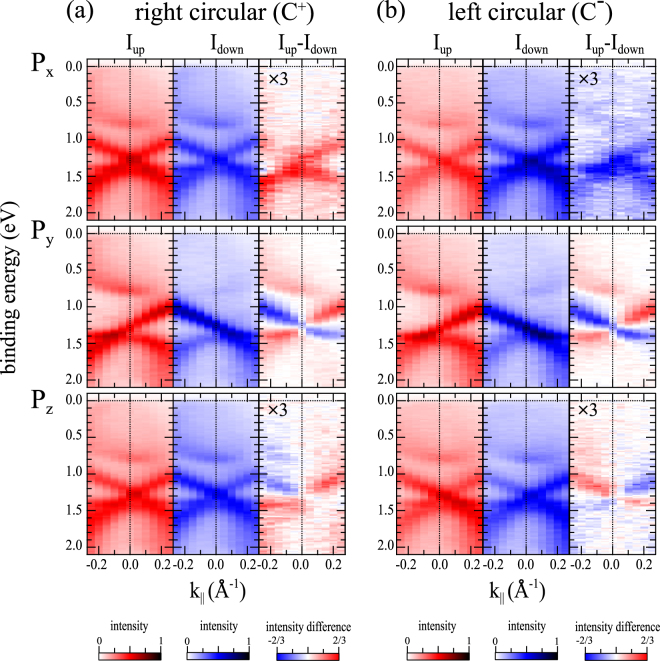
Spin-resolved photoemission intensities (1st and 2nd columns) and intensity differences between spin-up and spin-down (3rd columns) along $\overline{{\rm{\Gamma }}H}$ line obtained with right (**a**) and left (**b**) circular polarized light.

In Fig. 2, our spin-ARPES results are summarized as E vs k_||_ plots, where the spin-polarized photoemission intensities and their differences are given as colored contours. In the following, we focus on the spin differences for the three spin-polarization components in the right columns of Fig. 2(a) for C^+^ light and Fig. 2(b) for C^−^ light. Two major observations stand out and have to be explained:

(i) By switching the circular polarization, the sign of P_x_ and P_z_ is reversed but P_y_ is unchanged.
(ii) By reversing the sign of k_||_ or *θ*_e_, P_y_ and P_z_ change sign, while P_x_ is unchanged.

For measurements along $\overline{{\rm{\Gamma }}H}$, due to the m_*xz*_ mirror plane as shown in Fig. 1(d), the initial-state spin polarization is restricted to P_y_. As a consequence, the observed non-zero P_x_ and P_z_ spin components are caused by the photoemission process itself, which is described by the full dipole-transition matrix element including the final state. The following discussion focuses on the photoemission-induced effect for P_x_ and P_z_.

First, we discuss the origin of observation (i). It can be explained by considering the symmetry of the experimental geometry including the mirror plane m_x*z*_ as illustrated in Fig. 1(d). Upon reflection at the mirror plane, the circular polarization of the light is reversed, while the light incidence angle and the electron emission angle are unchanged. In addition, P_y_ is unchanged, while P_x_ and P_z_ change sign. As a consequence, P_x_ and P_z_ are expected to change sign upon switching the circular polarization of the light. This situation is generally realized in experiments, where the detection plane coincides with a mirror plane, which is the case for $\overline{{\rm{\Gamma }}H}$ on W(110).

Second, observation (ii) can be explained in the framework of a group-theoretical analysis of spin-dependent photoemission on the basis of dipole-transition matrix elements including final states of Σ_1_ representation (without spin-orbit interaction)^21^. In general, an initial state consisting of a mixture of spin-up and spin-down states may result in a spin polarization of the photo-emitted electron, which is different from the initial-state spin polarization.

Following the approach of ref.^21^, we can derive the total photoemission intensity *I* and the intensity differences ${I}_{{\rm{up}}}^{{\rm{x}},{\rm{y}},{\rm{z}}}-{I}_{{\rm{down}}}^{{\rm{x}},{\rm{y}},{\rm{z}}}=I{{\rm{P}}}_{{\rm{x}},{\rm{y}},{\rm{z}}}$. The orbital-dependent spin polarization of the initial states is considered, while the final states have Σ_1_ symmetry. The latter follows from the fact that there is no spin-orbit interaction at the detector (vacuum), spin-orbit coupling is very weak for electronic states with energies much higher than the Fermi level, and only even states can be detected.

1 $$I({{\rm{C}}}^{\pm })=si{n}^{2}{\theta }_{{\rm{p}}}{|{M}_{p\perp }^{\mathrm{(1)}}|}^{2}+\mathrm{(1}-co{s}^{2}{\varphi }_{{\rm{p}}}si{n}^{2}{\theta }_{{\rm{p}}}){|{M}_{p||}^{\mathrm{(3)}}|}^{2}+\mathrm{(1}-si{n}^{2}{\varphi }_{{\rm{p}}}si{n}^{2}{\theta }_{{\rm{p}}}){|{M}_{s}^{\mathrm{(4)}}|}^{2}$$

2 $$I{{\rm{P}}}_{{\rm{x}}}({{\rm{C}}}^{\pm })=2sin{\theta }_{{\rm{p}}}(\pm cos{\varphi }_{{\rm{p}}}{\rm{Re}}({M}_{p\perp }^{\mathrm{(1)}}{M}_{s}^{\mathrm{(4)}\ast })-sin{\varphi }_{{\rm{p}}}cos{\theta }_{{\rm{p}}}{\rm{Im}}({M}_{p\perp }^{\mathrm{(1)}}{M}_{s}^{\mathrm{(4)}\ast }))$$

3 $$I{{\rm{P}}}_{{\rm{y}}}({{\rm{C}}}^{\pm })=-\,2sin{\theta }_{{\rm{p}}}(cos{\varphi }_{{\rm{p}}}cos{\theta }_{{\rm{p}}}{\rm{Im}}({M}_{p\perp }^{\mathrm{(1)}}{M}_{p||}^{\mathrm{(3)}\ast })\pm sin{\varphi }_{{\rm{p}}}{\rm{Re}}({M}_{p\perp }^{\mathrm{(1)}}{M}_{p||}^{\mathrm{(3)}\ast }))$$

4 $$I{{\rm{P}}}_{{\rm{z}}}({{\rm{C}}}^{\pm })=\mp \,cos{\theta }_{{\rm{p}}}{\rm{Re}}({M}_{p||}^{\mathrm{(3)}\ast }{M}_{s}^{\mathrm{(4)}})-2sin2{\varphi }_{{\rm{p}}}si{n}^{2}{\theta }_{{\rm{p}}}{\rm{Im}}({M}_{p||}^{\mathrm{(3)}}{M}_{s}^{\mathrm{(4)}\ast })$$

*θ*_p_ and *ϕ*_p_ represent polar and azimuthal angles of the incident photons, respectively. In our experimental geometry, *ϕ*_p_ = 0°. As a consequence, only the first terms of eqs (2–4) are relevant for the discussion of the observed spin polarization. ${M}_{p\perp }^{\mathrm{(1)}}$ and ${M}_{p||}^{\mathrm{(3)}}$ (${M}_{s}^{\mathrm{(4)}}$) indicate the complex partial matrix elements for Σ_1_ and Σ_3_ (Σ_4_) orbital contributions in the initial state, excited by normal and parallel electric field vector components of *p*-polarized (*s*-polarized) light within circular polarized light, respectively. For normal electron emission and small *θ*_*e*_, we neglect photoemission contributions of Σ_2_ states because they are forbidden by selection rules at $\overline{{\rm{\Gamma }}}$^31^. From these equations, P_y_ originates from a mixing of Σ_1_ and Σ_3_ even-symmetry orbitals with respect to the *m*_*xz*_ mirror plane. This photoemission-induced effect of P_y_ is caused by only the *p*-polarized component of the light. The intensity difference for P_x_ (P_z_) is generated by a mixing of Σ_1_ (Σ_3_) even-symmetry orbitals and Σ_4_ (Σ_4_) odd-symmetry orbitals. The unexpected intensity differences for P_x_ and P_z_ are due to coherent superposition of *p*− and *s*−polarization components within the circular polarized light. In the experiment, we varied the photoelectron emission angle *θ*_*e*_ between −5° and +5°. Consequently, according to our set-up, the light incidence angle *θ*_p_ varies between 45° and 55°, which will not strongly influence the intensity differences. The variation of *θ*_*e*_ is taken into account in the complex partial matrix elements ${M}_{p\perp }^{\mathrm{(1)}}$, ${M}_{p||}^{\mathrm{(3)}}$, and ${M}_{s}^{\mathrm{(4)}}$.

**Figure 3:**
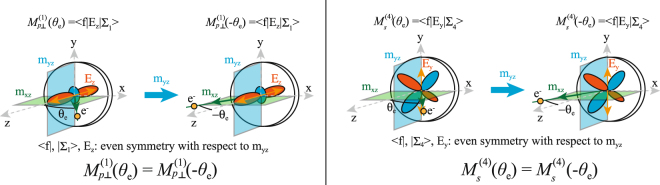
Symmetry of partial matrix elements ${{\rm{M}}}_{\perp }^{1}$ and ${{\rm{M}}}_{||}^{4}$ related to the mirror plane m_y*z*_. The partial matrix elements include initial state, final state and electric field vector of the light used for excitation of photoelectrons.

Next, we discuss the symmetry of the partial matrix elements in more detail, as illustrated in Fig. 3. The partial matrix elements contain the wave function of the final state, the electric field vector of the photons, and the wave function of the initial state with C_*2v*_ symmetry, namely, Σ_1_, Σ_3_, and Σ_4_. (In double-group representation, only one representation (Σ_5_) exists which can be decomposed into the four single-group representations). The surface with C_*2v*_ symmetry includes two mirror planes: m_*xz*_ and m_*yz*_. In our experimental geometry, final state, electric field vector components E_*z*_ and E_*y*_, and Σ_1_ and Σ_4_ initial states have even symmetry with respect to m_*yz*_. However, the photoelectron emission angle is reversed upon reflection at m_yz_. As a consequence, ${M}_{p\perp }^{\mathrm{(1)}}({{\rm{\theta }}}_{e})={M}_{p\perp }^{\mathrm{(1)}}(\,-\,{\theta }_{e})$ and ${M}_{s}^{\mathrm{(4)}}({{\rm{\theta }}}_{e})={M}_{s}^{\mathrm{(4)}}(\,-\,{\theta }_{e})$. For ${M}_{p||}^{\mathrm{(3)}}$, we can estimate the symmetry properties. In a simple model, a matrix element is the Fourier transform of the initial state’s orbitals; thus, it obeys the same even-odd properties in k-space as the orbital itself. Therefore, ${M}_{p||}^{\mathrm{(3)}}$ has odd symmetry with respect to m_yz_: ${M}_{p||}^{\mathrm{(3)}}({{\rm{\theta }}}_{e})=-\,{M}_{p||}^{\mathrm{(3)}}(\,-\,{\theta }_{e})$. By considering the *θ*_*e*_-dependent partial matrix elements, *I*P_x_ (*I*P_y_ and *I*P_z_) show even (odd) symmetry, with respect to *m*_yz_. Therefore, the intensity differences for P_z_ are reversed, when *θ*_*e*_ changes sign, while P_x_ does not change.

**Figure 4:**
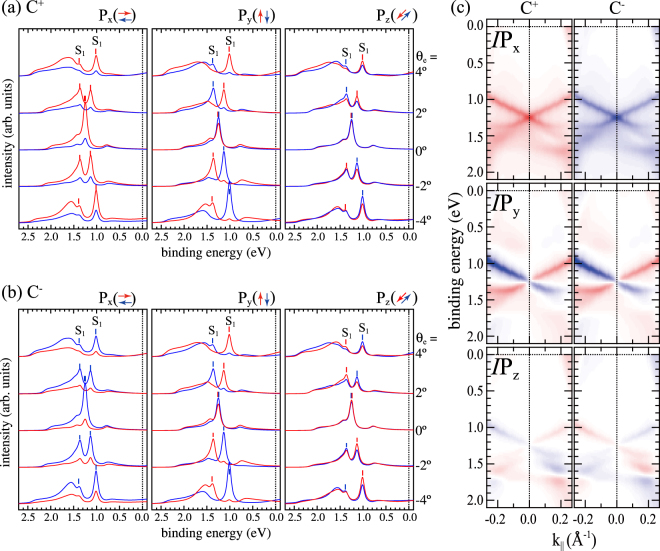
(**a**,**b**) Spin-resolved EDCs calculated within the one-step model of photoemission along $\overline{{\rm{\Gamma }}H}$, for P_x_, P_y_, and P_z_, and right and left circular polarized light. (**c**) Intensity differences between spin-up and spin-down for all spin components.

Figure 4 shows first-principles calculations within the relativistic one-step model calculation for W(110) along $\overline{{\rm{\Gamma }}H}$. The calculation includes the full photoemission process for excitation with circular polarized light and the geometrical set-up of our experiment. EDC’s for C^+^ and C^−^ light are presented for all three spin polarization components in Fig. 4(a,b), respectively. The intensity differences are shown in Fig. 4(c). We find good qualitative agreement between experimental and theoretical results, even details, such as spin-dependent intensities for P_*y*_ at *θ*_*e*_ = 0°, are well reproduced.

In conclusion, we have clarified the origin of circular-polarized-light-induced spin signals in photoemission results from the Dirac-cone-like surface state on W(110) with C_*2v*_ crystal symmetry. We observed non-zero spin polarization in all spin components P_x_, P_y_, and P_z_. In our case, for photoelectrons from the Dirac-like state on W(110) with *hν* = 43 eV, we obtained spin-polarization values of up to 20% for P_x_, 90% for P_y_, and 25% for P_z_. While P_y_ is dominated by the *intrinsic* spin polarization of the initial states, P_x_ and P_z_ are exclusively *extrinsic* and originate from the photoemission process. Our detailed analysis of the complex partial transition matrix elements shows how the orbital contributions of the initial state and the geometrical details of the experiment, such as light incidence angle and light polarization, and the crystal symmetry lead to the experimentally observed spin texture. Within limits, this approach opens the way for manipulating and controlling the spin polarization of photo-emitted electrons. The limits are given by the sample and its symmetry, the orbital characters of the respective electron states, and the experimental parameters. The challenge is to find optimum conditions for sample and experimental parameters that lead to maximum spin-polarization values.

The discussed mechanism of inducing spin polarization by circular polarized light is a general phenomenon depending on the specific orbital characters of the bands as well as geometrical parameters of the experiment. While the described test case of an occupied Dirac state at the metallic W(110) surface is not well suited for spin-selective transport, the general idea may be applied to optical-induced spin-polarized transport in optospintronic devices (see, e.g.,^35^). One may even speculate about spin-selective anisotropic transport phenomena by exploiting anisotropically dispersing states similar to the Dirac state on W(110)^31^.

## Methods

**Figure 5:**
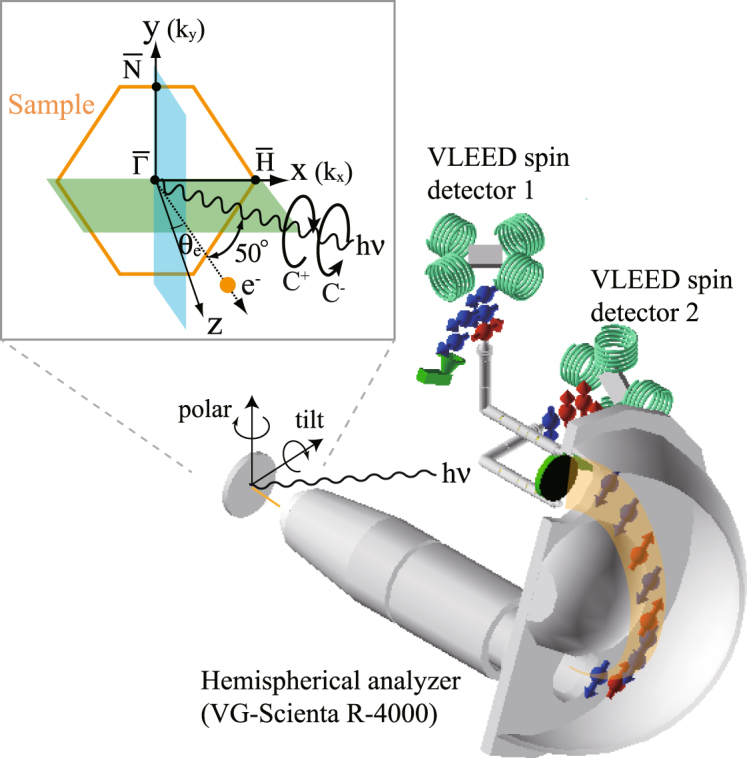
Schematic illustration of the experimental geometries of our spin-ARPES experiment (ESPRESSO).

A clean surface of W(110) was obtained and evaluated by the same procedure as described elsewhere^30,31^. The ARPES and spin-ARPES experiments were performed with synchrotron radiation generated by a quasi-periodic variable polarizing undulator at BL-9B at Hiroshima Synchrotron Radiation Center (HiSOR), equipped with highly efficient three-dimensional spin-polarization analysis of the ESPRESSO machine^36,37^. At BL-9B, the electric field vectors between left (C^−^) and right circular (C^+^) can be switched by changing the magnetic phase of the variable polarizing undulator. The angle of light incidence was 50° relative to the lens axis of the electron analyzer in all experiments as shown in Fig. 5. The spin-ARPES system with high angular- and energy-resolution can resolve all three spin polarization components: out-of-plane (P_z_) and in-plane (P_x_ and P_y_). The positive (negative) sign of spin polarization is parallel (antiparallel) to the arrows of the x, y, and z axes in the sample coordinate system. The emission angle *θ*_*e*_ of the photoelectrons is defined as positive (negative), when the surface normal is moved away from (toward) the light propagation vector. The overall experimental energy and angular resolutions of ARPES (spin-ARPES) at BL-9B were set to 50 meV (50 meV) and 0.3° (0.75°), respectively. All measurements have been performed at a sample temperature of 80 K.

The first-principles calculations include the photoemission process in the one-step model and are described in ref.^7^.

The datasets generated during and/or analysed during the current study are available from the corresponding author on reasonable request.
